# Human Gingiva-Derived Mesenchymal Stem Cells Ameliorate Streptozoticin-induced T1DM in mice *via* Suppression of T effector cells and Up-regulating Treg Subsets

**DOI:** 10.1038/s41598-017-14979-5

**Published:** 2017-11-10

**Authors:** Wei Zhang, Li Zhou, Junlong Dang, Ximei Zhang, Julie Wang, Yanming Chen, Jichao Liang, Dongqing Li, Jilin Ma, Jia Yuan, Weiwen Chen, Homayoun H. Zadeh, Nancy Olsen, Song Guo Zheng

**Affiliations:** 1 Expert Workstation and Division of Endocrinology, Qujing Affiliated Hospital of Kunming Medical University, Qujing, Yunnan Province, China; 2 Department of Clinical Immunology and Division of Endocrinology, The Third Affiliated Hospital of Sun Yat-sen University, Guangzhou, Guangdong Province China; 3 Division of Rheumatology, The Pennsylvania State University, College of Medicine, 500 University Drive, Hershey, 17033 PA USA; 4 Division of Nephrology, Zhejiang Traditional Chinese Medicine and Western Medicine Hospital, Hangzhou, Zhejiang Province, China; 5 Division of Periodontology, Diagnostic Sciences & Dental Hygiene, University of Southern California Ostrow School of Dentistry, Los Angeles, CA 90089 USA

## Abstract

There is yet no cure for type 1 diabetes (T1DM) so far. A significant body of evidence has demonstrated that bone marrow-derived mesenchymal stem cells (BMSCs) showed great potential in controlling T1DM. But there exists much difficulty in using BMSCs as a clinical therapy. We here test whether a new population of mesenchymal stem cells from human gingiva (GMSCs), which has many advantages over BMSCs, can delay or prevent progress of T1DM. GMSCs were adoptively transferred to multiple low-dose streptozotocin (STZ)-induced T1DM. Blood glucose levels and disease severities were analyzed. T cells subsets in blood, spleen and lymph nodes were detected dynamically by flow cytometry. GMSC distribution was dynamically analyzed. We found that infusion of GMSCs but not fibroblast cells significantly controlled blood glucose levels, delayed diabetes onset, ameliorated pathology scores in pancreas, and down-regulated production of IL-17 and IFN-γ in CD4^+^ and CD8^+^ T cells in spleens, pancreatic lymph nodes (pLN) and other lymph nodes. GMSCs also up-regulated the levels of CD4^+^ Treg induced in the periphery. Mechanismly, GMSCs could migrate to pancreas and local lymph node and function through CD39/CD73 pathway to regulate effector T cells. Thus, GMSCs show a potential promise in treating T1DM in the clinic.

## Introduction

T1DM is a chronic autoimmune disease in which insulin-secreting pancreatic beta cells are attacked and destroyed by autoreactive T cells. Auto-antibodies like GAD65, insulinoma-associated protein 2 (IA-2), and tyrosine phosphatase or zinc transporter (ZnT8) to insulin are much higher in most T1DM patients^1^. Over the past 40 years, the incidence of childhood T1DM worldwide has increased by 3–5% annually^2^. Insulin is the main treatment for T1DM patients, and human islet transplantation also has emerged as a treatment, since insulin may cause severe hypoglycemia and some patients are not sensitive to insulin. But these therapeutic approaches have no effect on the autoimmune process and cannot alleviate the pathogenesis, so that patients develop long-term complications eventually. Therefore, novel approaches to cure T1DM are badly needed.

Mesenchymal stem cells (MSCs) are multipotent progenitor cells, which can proliferate in an *in vitro* condition, differentiate into bone, cartilage, and adipose tissues^3^. MSCs also display profound immunomodulatory and anti-inflammatory capabilities. These cells can inhibit the proliferation and activation of T effector cells, as well as support induction of CD4^+^ Tregs^4–6^. Indeed, MSCs have been used to reduce the burden of a variety of autoimmune diseases, including graft-*versus*-host disease^7,8^, rheumatoid arthritis^9^ and systemic lupus erythematosus^10^. BMSCs have been considered as a potential clinical treatment, but difficulties with harvest and dysfunction of autologous BMSC as well as potential for tumorigenesis have limited their clinical application^11^.

Gingiva-derived mesenchymal stem cells (GMSCs) have emerged as a novel MSC population that not only have the similar biological features, but also display some advantages over other MSCs^12–14^. GMSCs are easy to isolate, proliferate more rapidly than BMSCs, are stable even at higher passages numbers, and most importantly, not tumorigenic^11^. GMSCs, but not fibroblast cells, which are always used as control cells because of having similar morphogy with GMSCs, have been showed to differentiate into cartilage, bone and adipose and have significant therapeutic effects in experimental colitis^15^, rheumatoid arthritis^16^, and xeno-GVHD^17^, but the utilization of GMSCs for the treatment of diabetes has never been explored.

Studies in animal models and clinical trials have suggested involvement of imbalance of Th17 cells and Tregs in the pathogenesis of T1DM. Th17 cells are up-regulated and Tregs have functional and/or quantity defects in T1DM patients^18,19^. The early augmentation of IFN-γ, TNF-α, IL-6 and IL-17 levels in pancreatic tissue correlates with pancreatic islet inflammation and beta cell damage. Anti-IL-17 antibody has been shown to reduce T-cell infiltration of islets, decrease autoantibody levels and increase the frequency of Treg cells in NOD mice^20,21^. Tregs show potential as a treatment for diabetes in NOD mice and in human patients^22,23^. It is not clear whether GMSCs could ameliorate diabetes *via* suppressing IL-17 and IFN-γ production and enhancing Tregs function or numbers.

Current studies indicated that CD39/CD73 might control cellular immune response by conversion of ADP/ATP to AMP and AMP to adenosine, respectively, thus driving a shift from an ATP-driven proinflammatory environment to an anti-inflammatory milieu induced by adenosine^24^. CD39 and CD73 were also shown coexpressed on multipotent mesenchymal stromal cells and the inhibition of T cell proliferation and function was mediated by CD39/CD73 expression and adenosine generation^25,26^. Indoleamine 2,3-dioxygenase (IDO) which catalyzes conversion from tryptophan to kynurenine has recently been identified as another major immunosuppressive effector pathway^27^. Studies from our group showed that human GMSCs also highly expressed CD39 and CD73 and they could significantly inhibit collagen-induced arthritis^16^ and xeno-GVHD^17^ *via* CD39/CD73 and/or IDO signals although it is still unknown whether these signal pathways contribute to T1DM suppression mediated by GMSCs.

STZ, a toxin that binds to the GLUT2 receptor on pancreatic beta cells, has been used for decades to induce diabetes in rodent models^28^. The multiple, low-dose STZ approach, in contrast with a single high dose STZ injection, induces distortion of the islet architecture in conjunction with mononuclear cell infiltration and apoptosis of beta cell, thus provides an environment in which islet autoantigens can be processed and presented by infiltrating APCs to autoreactive T cells that have escaped thymic deletion^29^ and immune cell mediated injury by autoreactive T cells is thought to be the dominant pathogenic mechanism^30^.

In present study, we have used STZ-induced T1DM mice and found GMSCs but not control cells significantly delayed T1DM onset. Additionally, GMSCs need CD39/CD73 signal to suppress T1DM, providing a potential GMSCs-based cell therapy in clinical applications for patients with diabetes and other autoimmune diseases.

## Results

### Phenotypic and functional characteristics of GMSCs

**Figure 1:**
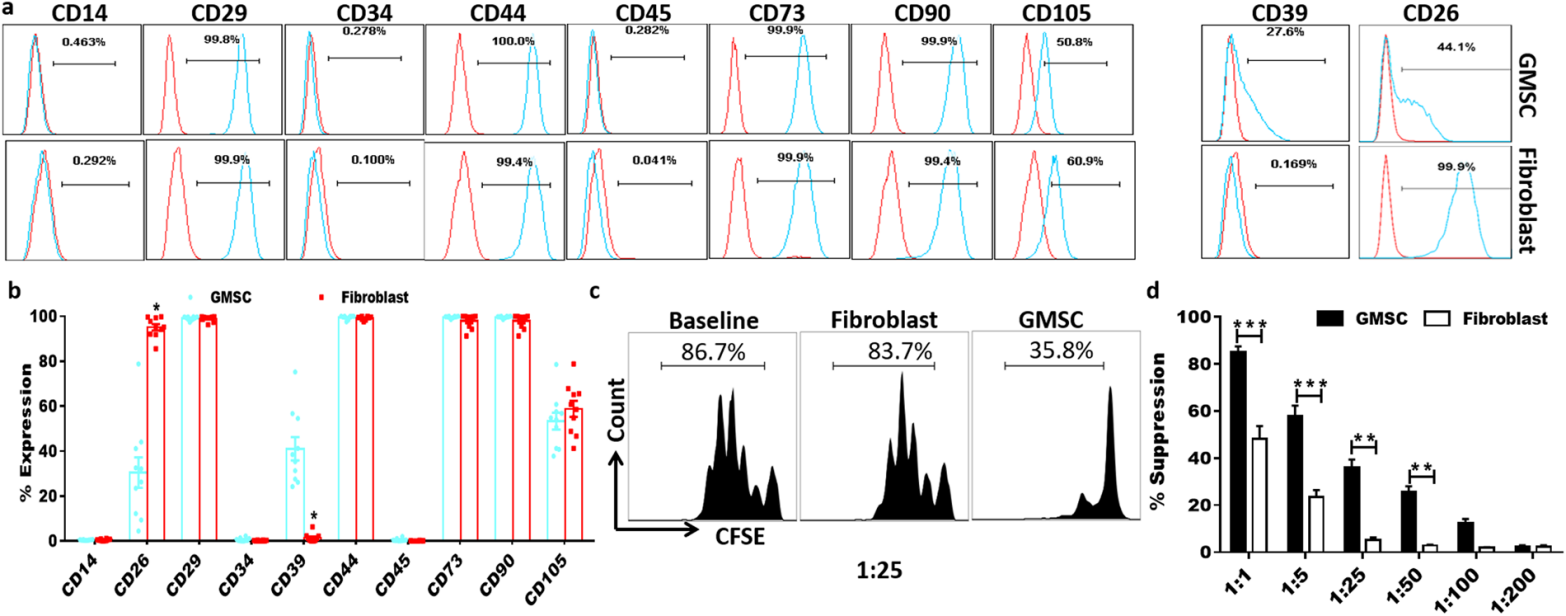
Phenotypic and functional characteristics of GMSCs. GMSCs and fibroblast cells were stained with a series of sufure makers and used for *in vitro* suppressive assay. (**a**) Representative flow data showed related phenotypes of GMSC and fibroblast cells. (**b**) Statistics analysis of each marker expression in two cell types (GMSCs were collected from ten donors and fibroblast cells from commercial resource). (**c** and **d**), Representative flow data showed that only GMSCs have suppressive function on mouse T cells even in the ratio of 1:25 (GMSCs or fibroblast cells: CD25^−^ T cells) (**c**) and statistic analysis (**d**) Data are presented as the mean ± SEM from 4 independent experiments. ***P < 0.001, **P < 0. 01.

GMSCs is one subset of MSCs that shares similar morphology and some phenotypic features with fibroblast cells. As shown in Fig. 1, while both GMSC and fibroblast cells similarily expressed CD29, CD44, CD73, CD90 and CD105 and did not express hemeopoietic cell markers such as CD14, CD34 and CD45. Nonetheless, GMSCs had a higher expression of CD39 while CD26 was highly expressed on fibroblast cells, indicating they are different cell populations (Fig. 1a and b)^31^. Additonally, GMSC but not fibroblast cells potently suppressed T cell proliferation (Fig. 1c and d), demonstrating they have a different biological activity.

### GMSCs ameliorated the development of T1DM in mice

**Figure 2:**
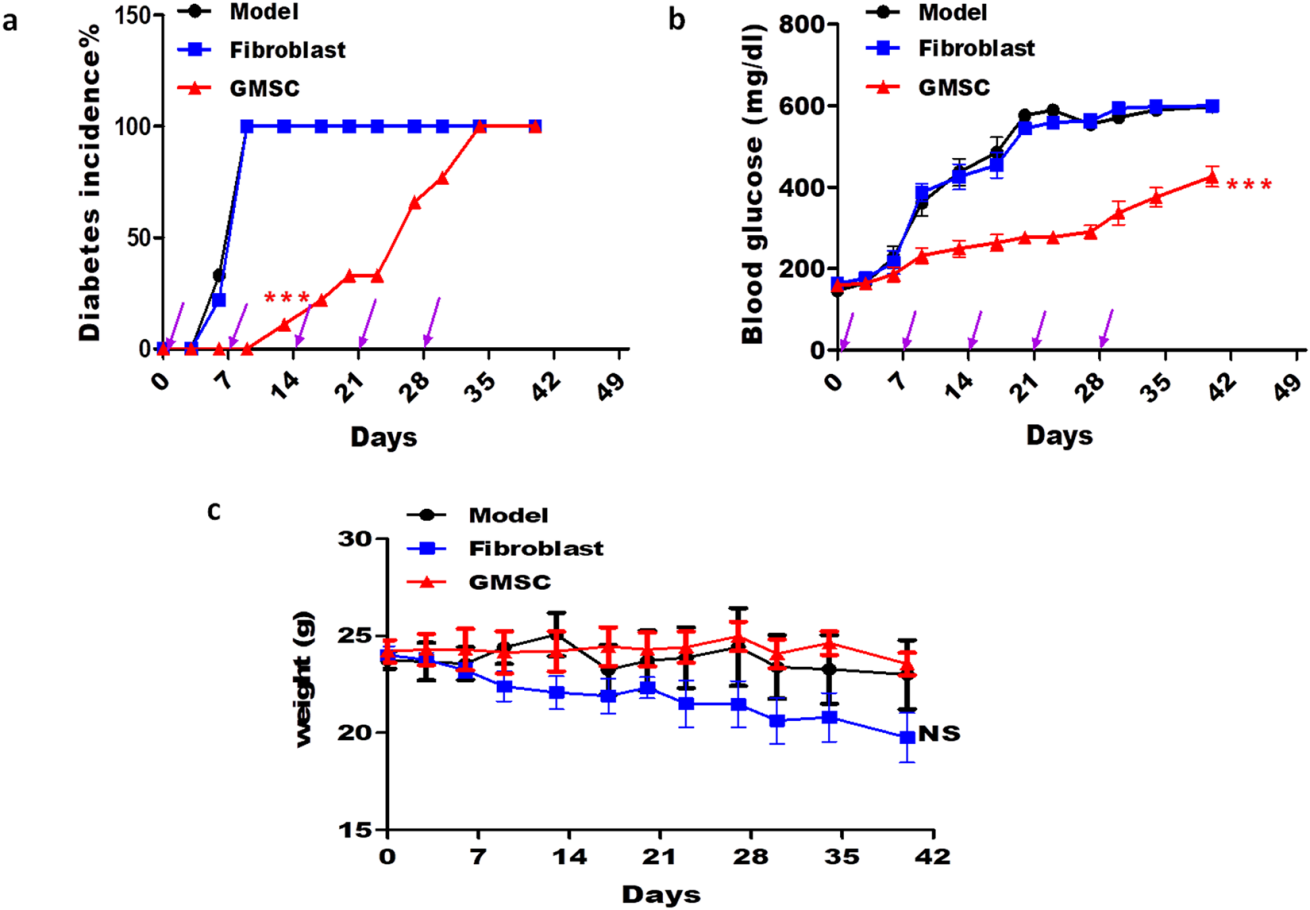
GMSCs ameliorated the development and severity of T1DM in mice. T1DM was induced in male C57BL/6-FoxP3^gfp^ mice using multiple low doses of STZ injection, 1 × 10^6^ GMSCs or human skin fibroblast cells were injected into mice *via* intraperitoneal route on days 0, 7, 14, 21, 28. (**a**) Incidence of diabetes. Blood glucose concentration exceeding 300 mg/dL in two consecutive daily measurements was considered diabetes. (**b**) Non-fasting blood glucose of mice in three groups. (**c**) Body weight in different groups. Data are presented as the mean ± SEM from two independent experiments (n = 5). ***P < 0.001, *versus* the fibroblast or model groups.

To investigate the effect of GMSCs in experimental diabetes, we used STZ-induced T1DM model in male C57BL/6-FoxP3^gfp^ mice. We chose male mice because rodents were reported to show a substantial gender difference in STZ sensitivity with male mice and rats tend to be more susceptible to STZ-induced diabetes compared to little or no response in female mice and severe hyperglycemia was present in male mice receiving identical doses^32^. We wanted to use flow cytometry to dynamically test Treg cell numbers and phenotype in blood by tail vein bleeding, so we used a Foxp3-GFP-reporter mice, which required quite little lymphocytes than other methods. Diabetes onset was markedly delayed following GMSCs treatment, although GMSCs treatment did not completely prevent the onset of diabetes eventually (Fig. 2a). GMSCs significantly diminished blood glucose levels compared with the model and fibroblast treatment groups (P < 0.001)(Fig. 2b). The body weight of the three groups were monitored, and showed that GMSCs could inhibit weight loss, but there was no significant difference among three groups (P > 0.05) (Fig. 2c). Thus, we show that GMSCs transfer could delay T1DM onset and ameliorate disease severity.

### Decreased severity of insulitis following treatment with GMSCs

**Figure 3:**
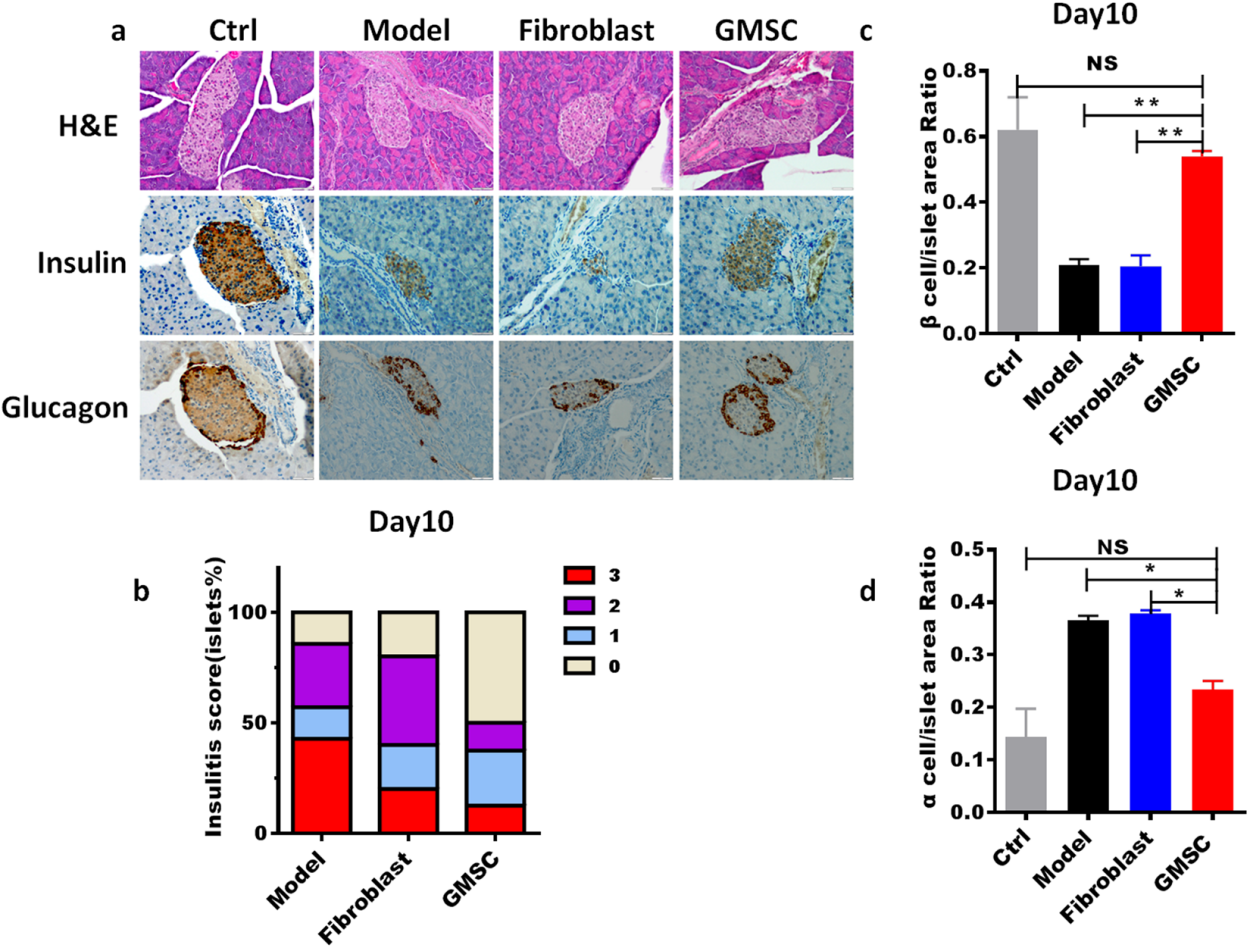
GMSCs decreased the severity of insulitis in mice model of T1DM. Pancreas were harvested on day 10, fixed in 10% formalin, embedded in paraffin. Six sections per pancreas, at least three individual pancreases from each group, were evenly sectioned and separated by 200 μm, stained with H&E, anti-insulin and anti-glucogon antibodies. (**a**) Hematoxylin and eosin staining and immunohistochemistry examination of pancreas from three groups. (**b**) Insulitis scores of pancreas on day 10. (**c**,**d**) Insulin-positive beta cells and glucogon-positive alpha cells in islets area were revealed by immunohistochemistry using the anti-insulin and anti-glucogon antibody on day 10. Data are presented as the mean ± SEM from two independent experiments using at least three individual pancreases from each group. *P < 0.05, **P < 0.01, *versus* the fibroblast or model groups.

Histological examination revealed that islets from GMSCs-treated mice exhibited bigger size than other two groups (Fig. 3a). GMSCs treated mice also showed higher proportions of insulin-positive beta cells (Fig. 3c) and lower proportions of glucogon-positive alpha cells (Fig. 3d) by immunohistochemistry using the anti-insulin and anti-glucagon antibodies on day 10. Insulitis score was significantly decreased in GMSCs treated mice compared with the model and fibroblast groups (Fig. 3b). We noted that GMSCs treatment could not prevent diabetes onset, insulitis and alpha/beta proportion has also no difference among three groups on day 30 (Supplementary Fig. S1).

### GMSCs down-regulated IL-17 and IFN-γ expression on both CD4^+^ and CD8^+^ T cells in STZ-induced T1DM model

**Figure 4:**
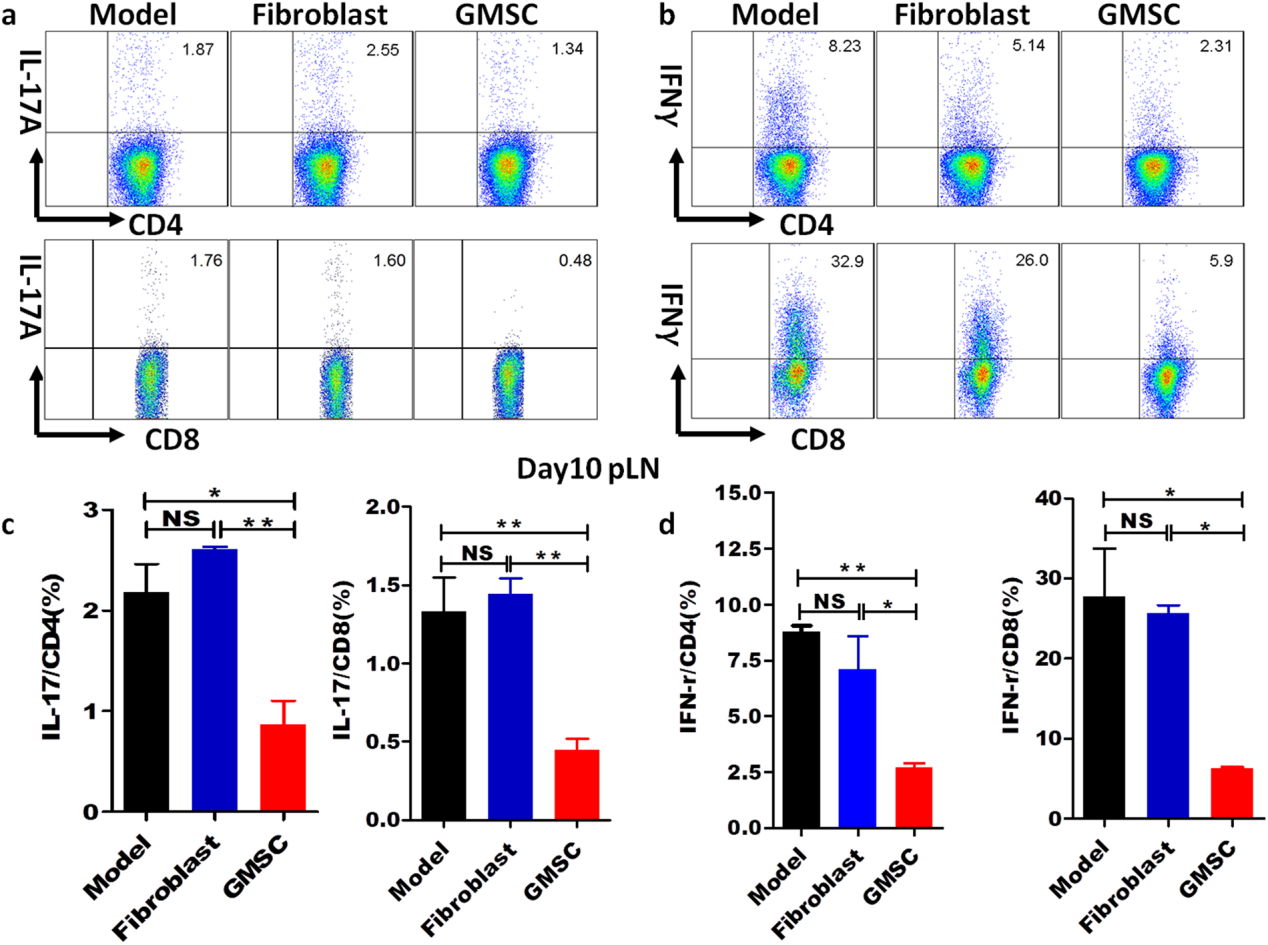
GMSCs down-regulated IL-17 and IFN-γ expression on CD4^+^ and CD8^+^ T cells in STZ-induced T1DM model. T1DM was induced using multiple low dose STZ injection and 1 × 10^6^ GMSCs or fibroblast cells were injected into mice *via* intraperitoneal route on days 0, 7, 14, 21, 28. pLN was harvested on day 10. Lymphocytes were isolated and then stimulated *in vitro* with PMA (50 ng/ml) and ionomycin (500 ng/ml) for 5 hours, with brefeldin A (10 μg/ml) added in the last 4 hours, and intracellular expression of IFN-γ and IL-17 on CD4^+^ and CD8^+^ T cells was analyzed by flow cytometry. (**a**,**b**) Representative flow data of IFN-γ and IL-17 expression gated on CD4^+^ T cells and CD8^+^ T cells in draining LN (pLN). (**c**,**d**) Expression of cytokines, including IFN-γ, IL-17 on CD4^+^ T and CD8^+^ T cells in the draining LNs of T1DM mice. Data are presented as the mean ± SEM from two independent experiments (n = 5). *P < 0.05, **P < 0.01, *versus* the fibroblast or model groups.

We next investigated the mechanisms underlying the decreased severity of diabetes following administration of GMSCs. Interleukin-17 (IL-17) has been associated with the pathogenesis of T1DM and our *in vitro* study found that GMSCs had the ability to inhibit Th17/Tc17 cells differentiation^16^, so we wondered whether GMSCs could suppress IL-17 expression in T1DM. Consistently with our *in vitro* study, we found that GMSCs significantly reduced the percentages of Th17 cells and Tc17 cells in pLN of mice with T1DM on day 10 after STZ induction compared with model, while fibroblast conversely had the tendency to increase Th17/Tc17 cells (Fig. 4a and c). In addition to IL-17 production, we also analyzed IFN-γ expression on T cells, since T1DM is considered a Th1 cells-mediated disease^33^. We also observed a suppressive function of GMSCs on IFN-γ expression on both CD4^+^ T cells and CD8^+^ T cells (Fig. 4b and d). IL-17 and IFN-γ expression on T cells in spleens and other lymph nodes were also decreased in GMSC treated group. These effects were consistent in spleens and other lymph nodes on day 30 after STZ induction (Supplementary Fig. S2). Thus, GMSCs delayed and treated diabetes at least partly through suppressing proliferation and activation of autoreactive T subsets, like Th17, Tc17, Th1, and Tc1 cells.

### GMSCs also promoted Treg production in STZ-induced T1DM mice model

**Figure 5:**
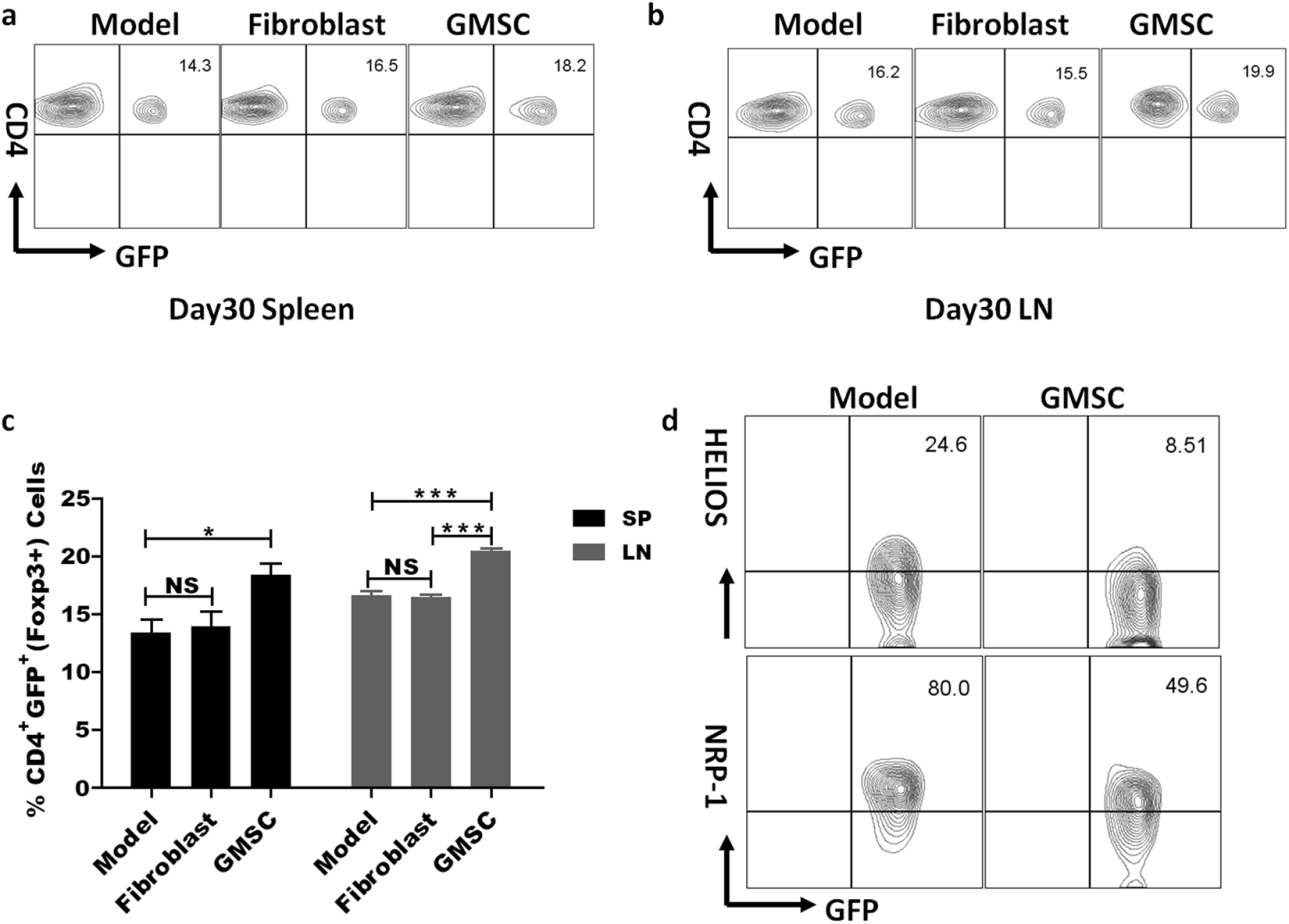
GMSCs up-regulated Treg production in STZ-induced T1DM mice model. Foxp3^gfp^ reporter C57BL/6 mice were injected with GMSCs (1 × 10^6^). CD4^+^ Foxp3^+^ (GFP^+^) Treg frequency was counted in the spleens and LN on day 30 after GMSC injection. (**a**.**b**) Representative flow data of CD4^+^ Foxp3^+^ frequency in day 30 spleen and LN in each groups. (**c**) statistics analysis of CD4^+^ Foxp3^+^ (GFP^+^) Treg frequency in slpeens and LN on day 30. Data are representative of two separate experiments and mean ± SEM of each group was shown (n = 3). *P < 0.05, ***P < 0.001 *versus* the control groups. (**d**) GMSCs increased the frequency of Helios^-^ Foxp3^+^ and Nrp-1^−^ Foxp3^+^ iTregs in T1DM mice model. Representative flow data of the Helios and Nrp-1 expression in LN. Cells were gated on CD4^+^ GFP^+^ cells.

Evidence has showed that Tregs are defective in numbers and/or function in animal model of T1DM and T1DM patients, so we tried to study the effects of GMSCs on Tregs *in vivo*. GMSCs treated group had a significantly larger number of CD4^+^ Treg cells in spleens and LNs than model and fibroblast groups on day 30 (Fig. 5a,b and c). According to previous studies, Nrp-1 and Helios could be used as specific cell markers to distinguish natural Tregs (nTregs) from induce Tregs (iTregs)^34,35^, we then tried to figure out whether these up-regulated Tregs originated from nTregs subsets or were induced by GMSCs *in vivo*. As shown in Fig. 5d, the majority of Tregs in GMSCs-treated mice were Helios negative and had a decrease of Nrp-1 expression compared with control groups, suggesting that GMSCs may induce the generation of iTregs *in vivo* rather than expand endogenous nTregs. CD4^+^ Tregs circulating in the peripheral blood and those residing in pLN of T1DM mice among the three groups had no statistical differences (Supplementary Fig. S3). As to the newly identified Treg cell population^36^, we found that GMSCs did not affect CD8^+^ CD103^+^ CD122^+^ CD28^−^ GFP^-^ Treg cells in the diabetes model (data not shown).

### Injected GMSCs migrated to pancreas and local lymph nodes

**Figure 6:**
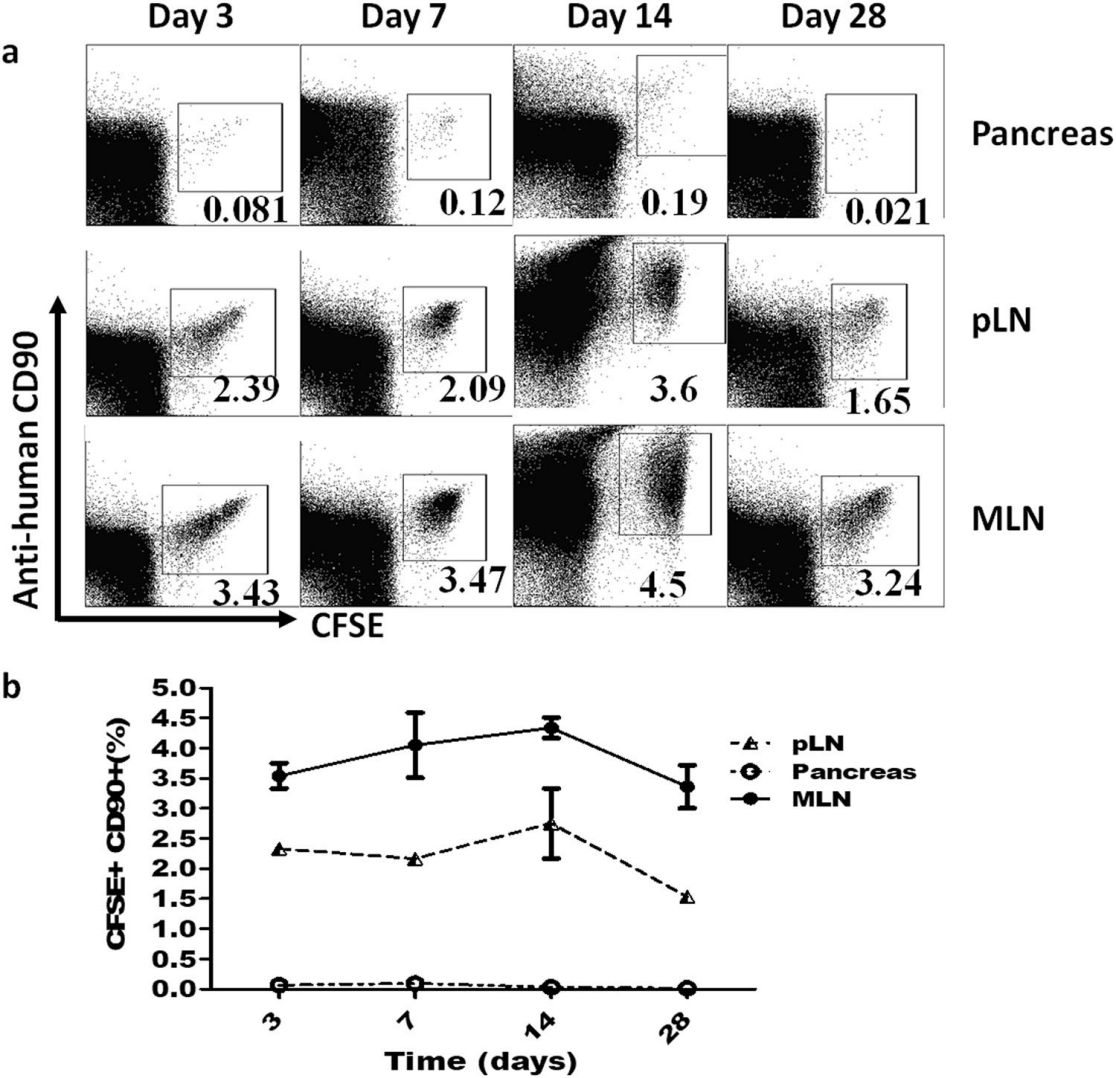
Intraperitoneal injected GMSCs migrated to pancreas and local lymph nodes. GMSCs were labeled with CFSE before *i.p*. injection and GMSCs organ distribution and engraftment was then examined at different time points post-injection by flow cytometry. (**a**) Representative flow data (%) showed that *i.p*. injected GMSCs could home to mesenteric lymph nodes (MLN) and pancreas lymph node (pLN) in a large percentage and the target organ—pancreas, regardless of a lower percentage at different time points. (**b**) Statistic chart for (**a**). Each time point had two mice and mean ± SEM of each time point in different tissues was shown. Experments were repeated twice with the similar results.

Previous studies suggested that therapeutic efficacy of MSCs was greatly dependent on their ability to produce juxtacrine or paracrine factors and migration of MSCs to the diseased organs/tissues was required for the juxtacrine effects^37^. We therefore also investigated the ultimate fate of the GMSCs following intraperitoneal injection. The intravital fluorescent dye, carboxyfluorescein diacetate succinimidyl ester (CFSE), which does not interfere with cytotoxic function, has been used extensively for studying lymphocyte migration and study reported that adoptively transferred lymphocytes labeled with CFSE could be indentified *in vivo* at least 2–3 months after injection^38^. To this end, we labeled GMSCs with CFSE before *i.p*. injection. GMSCs organ distribution and engraftment was then examined at different time points post-injection by flow cytometry. The results showed that *i.p*. injected GMSCs mainly migrated to lymph nodes (Fig. 6a and b) but barely exsisted in brain, lung, kidney or liver (data not shown) on day 3, 7, 14 and 28. Interestingly, GMSCs homed to pancreas lymph nodes in a large percentage and the target organ—pancreas regardless of a lower percentage at different time points (Fig. 6a and b).

### GMSCs functioned through CD39/CD73 pathway to regulate T effector cells

**Figure 7:**
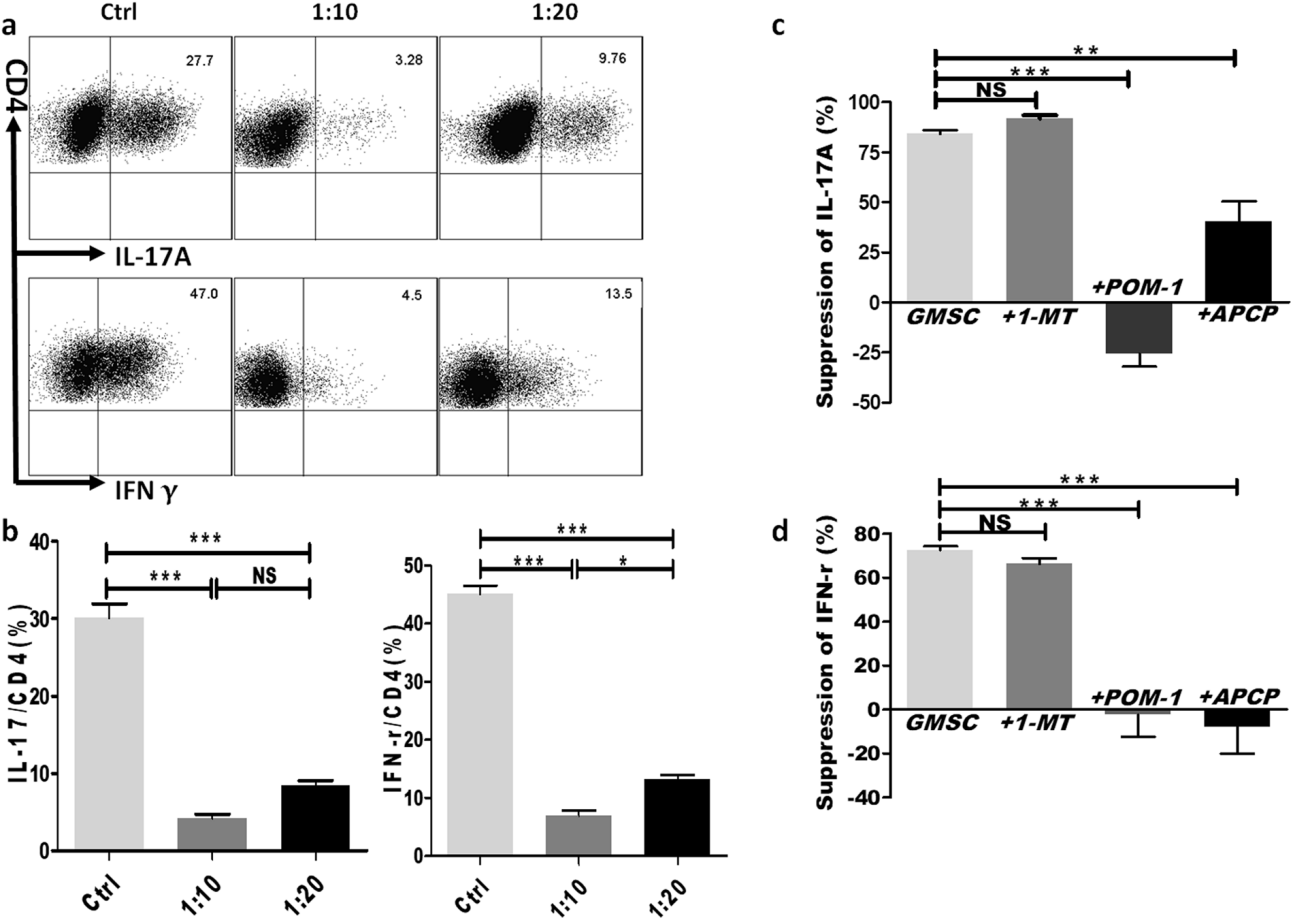
GMSCs inhibited the differentiation of mouse CD4^+^ T effector cells through CD39/CD73 signals. (**a**) Naïve CD4^+^ T cells were cultured without (Ctrl) or with GMSCs (GMSCs:naïve CD4^+^ T cells = 1:10 or 1:20) for 3 days under Th17 or Th1 cell-polarization conditions. Expression of intracellular cytokines (IL-17 and IFN-γ) in each T helper cell subset was analyzed by flow cytometry (representative data shown); (**b**) statistics analysis for (**a**); (**c** and **d)** GMSCs were pre-treated with solube factors (APCP, POM-1, 1-MT; DMSO or isotype were used as controls) for 24 hours, then GMSCs were washed completely with PBS in well, and suppression of CD4^+^ T cell differentiation was determined (GMSCs:naïve CD4^+^ T cells = 1:20). Bars showed the mean ± SEM of 3 separate experiments. *P < 0.05, **P < 0.01, ***P < 0.001.

We used an *in vitro* naïve CD4^+^ T cells differentiation experiment to explore the possible mechanisms responsible for GMSCs-mediated suppression of T effector cells in STZ-induced T1DM mice model. Same as previous reports, GMSCs significantly inhibited murine CD4^+^ T cells differentiation into Th1 or Th17 cells in a dose-dependent manner (Fig. 7a and b). To exclude the possible effect of different solube factors on T cells, we pre-treated GMSCs with CD39, CD73 and IDO inhibitor, respectively and then co-cultured GMSCs with T cells in an inhibitor-free system for 3 days. Results showed that after CD39 or CD73 inhibitor but not 1-MT treatment, GMSCs almost completely lost their immunosuppression on T effector cell differentiation, if any, this even lead a tendency to up-regulate IL-17 or IFN-γ expression (Fig. 7c and d), suggesting a vital role for CD39/CD73 signals in mediating the immunoregulatory function of GMSCs.

## Discussion

MSCs are multipotent progenitor cells, which can be found in bone marrow, adipose tissue, umbilical cord blood, the placenta of humans and human gingival tissues^39^. A decade of experiments has established that MSCs can inhibit the proliferation and function of T and B cells, NK cells, as well as promote the expansion of CD4^+^ Tregs^4–6^. MSCs-mediated immunosuppression ability is also associated with inhibition of pro-inflammatory cytokine induction^40^. Results of *in vivo* administration of MSCs in autoimmune diseases is consistent with *in vitro* studies^41,42^. MSCs as a therapy for T1DM has been studied before^42,43^. BMSC is the most intensively studied type of MSCs, however, there are substantial weaknesses of BMSC clinical applications: 1) an invasive process is needed to obtain BMSC; 2) BMSCs are not stable after multi cycle proliferation; 3) BMSCs are dysfunctional in patients with autoimmune diseases^44,45^, use of autologous BMSC is unlikely to be feasible; 4) BMSCs have a tumorigenesis risk^46–48^.

GMSCs have many advantages over BMSCs as this population is abundant and more stable than BMSC as well as not having tumorigenic potential^11^. GMSCs have been showed to have significant therapeutic effect in experimental colitis^15^ and rheumatoid arthritis^16^. Our recent study demonstrated that GMSC can suppress human cell-mediated xeno-GVHD^17^, implying a potential for clinical translation. In the current study, we have provided a line of new evidence that GMSCs administration can be a potential therapy for autoimmune diabetes since they could delay STZ-induced diabetes onset, control blood glucose level, prevent weight loss, decrease insulitis score and preserve beta cells. Moreover, as far as we know, our group is the first to show that GMSCs have such function on diabetes.

T1DM, which was previously thought to be a Th1-mediated autoimmune disease, is now considered also associated with Th17 cells^49^. IL-17 expression in the islet correlates with insulitis in spontaneous autoimmune diabetes model in nonobese diabetic (NOD) mice^50^. Inhibition of Th17 cells significantly suppressed development of diabetes^51^. IL-17 deficiency delayed onset of diabetes, with reduced insulitis in NOD mice, and ameliorates STZ-induced diabetes^52,53^. Adoptive transfer of *in vitro* differentiated islet antigen specific Th17 cells to immunodeficient mice can lead to rapid onset of diabetes with extensive insulitic lesions^50^. In addition to Th17 cells, CD8^+^ T cells that are characterized by the expression of the transcription factor retinoic acid-related orphan receptor (RORγt) and the production of IL-17, termed Tc17 cells, have recently been identified^54^. These cells have a role in diabetes pathogenesis, since percentages of circulating Tc17 cells in children with new-onset T1DM was increased^55^ and a role in diabetes was also demonstrated in a murine model^56^. All these findings clearly highlight the major role of IL-17 and IFN-γ in the development of diabetes. In accordance with these findings, our results showed that GMSCs can suppress mouse naïve CD4^+^ T cells differentiate into Th17 and Th1 cells *in vitro*. In the STZ-induced T1DM mice model, GMSCs could significantly inhibit IL-17 expression on CD4^+^ T cells and CD8^+^ T cells compared with model and fibroblasts groups. We also observed an gorgeous effect of GMSCs on IFN-γ expression, which was consistent with other reports^16^. So, it can be illustrated that effects of GMSCs on the diabetes model may be partly mediated by suppressing IL-17 and IFN-γ expression on autoreactive T cells.

MSCs are reported to increase the percentages of regulatory T and B cells^16,57,58^. In the present study, although we did not see any significant difference in CD4^+^ Tregs number in blood or pLN, we did find an increased number of CD4^+^ Foxp3^GFP+^ Tregs in GMSCs treatment group in spleen and MLN. Through analysis of the expression of nTregs cell markers, Nrp-1 and Helios, we found that GMSCs mainly induced iTregs rather than expanded nTregs thus resulting in up-regulation of Tregs subsets. Similar results have been reported in other models^16^. We recently identified a new CD8^+^ Treg cell population that requires CD103 for their development and function but are independent of the cytotoxic effect^59^. In this study we also analyzed CD8^+^ CD103^+^ CD122^+^ CD28^−^ GFP^-^ Treg cells in peripheral blood, spleen, MLN and pLN of three groups. We did not observe significant augmentation of CD8^+^ Tregs. Thus, GMSCs mainly function through inducing CD4^+^ Treg subsets, but not by affecting CD8^+^ Tregs, in delaying T1DM.

Number of therapeutic cells that can migrate and colonize at the injury site is a decisive prerequisite for the success of cell-based therapy. In line with other reports, which showed that *i.p*. injected MSCs engrafted more to inflamed colon in a DSS-induced colitis mouse model but showed fewer trapped cells in lung, liver and spleen compared to *i.v*. injection, thus resulting in better experimental colitis recovery^60^, our results showed that *i.p*. injected GMSCs barely existed in lung, liver, brain or kidney but mostly homed to lymph nodes, including pancreas lymph nodes and somehow to pancreas at different time points, which could attribute to the immunosuppressive role of GMSCs in T1DM model.

The immunoregulatory function of murine multipotent mesenchymal stromal cells, human BMSCs and regulatory T cells all have been reported to be mediated at least partially through CD39/CD73 signal pathways^25,26,61,62^. Studies from our own group also reported a CD39/CD73 dependent immunomodulatory function of human GMSCs^16,17^. In accordance with these reports, we found that GMSCs, which highly expressed CD39 and CD73, could inhibit T effector cells differentiation *in vitro* and this function was significantly abrogated by CD39 or CD73 inhibitor treatment, which could also be a mechanism of the *in vivo* suppression of GMSCs on T1DM. However, IDO was not significantly involved in this suppression. It is likely because immunosuppressive properties of IDO by MSCs could be affected by specific microenvironments^62^, expression of IDO is not constitutive but could be induced by IFN-γ and induction of functional IDO activity required MSC activation^27^.

In this study we only observe a delay but not complete prevention of diabetes onset after GMSCs treatment. It can not be explaned by a too short life span of GMSCs *in vivo*. Using tracking experiments, we can clearly observe the distribution of GMSCs even at day 28. It is possible that GMSCs might need a sufficient local concentration to exert their effect *in vivo*. Although GMSCs could migrate to target organs, the percentages of GMSCs in pancreas could be too low to exert an efficient immunosuppressive effect. Additionally, the *in vivo* inhibitory effect of GMSCs could be overcome by a high level of inflammation in T1DM model. As GMSCs were hardly detected in blood, lung and liver, and diabetes was a multiorgan-associated disease, the proinflammatory cytokines production in these organs might not be suppressed by GMSCs, and if these proinflammatory cytokines accumulated to a certain extent, it could offset or even overcome the immunosuppressive effect of GMSCs.

Based on evidence presented above,we conclude that GMSCs have the ability to migrate to pancreas and local lymph nodes and alter the imbalance between Tregs and T effector cells thus ameliorate STZ-induced T1DM in mice model. Mechanismly,GMSCs could function through CD39/CD73 pathway to regulate effector T cells. Our findings serve as evidence supporting that GMSCs, a unique population of MSCs, which has similar function with but much advantage over BMSCs, are a promising cell source for stem cell-based therapies of autoimmune diabetes and other autoimmune and inflammatory diseases.

## Materials and Methods

### Mice

Wild-type C57BL/6 (male, 6–8 week old, 20–26 g body weight) mice were obtained from Jackson Laboratory. C57BL/6-FoxP3^gfp^ mice were generously provided by Dr. Talil Chatilla (University of Southern California, Los Angeles). All animals were housed in SPF system in a temperature (72 ± 3 °F) and air (50 ± 20% relative humidity)-controlled room with a 12 h light-dark cycle and were given standard diet and tap water. All experiments using mice were performed in accordance with protocols approved by the Institutional Animal Care and Use Committee at the Third Affiliated Hospital of Sun Yat-sen University.

### GMSCs isolation, culture and identification

Ten samples of healthy human gingival tissues (from healthy Asian male or female who experienced an operation of wisdom tooth extraction surgery, aged between 20 and 30 years old, without any autoimmune or inflammatory diseases, all from Guangdong Province of China) were obtained as remnant or discarded tissues following routine dental procedures at the Third Affiliated Hospital of Sun-Yat-sen University, in accordance with the ethical standards and with the approval of Institutional Review Board protocol of Sun Yat-sen University. Informed consent was obtained from all participating human subjects for the collection of fresh tissue. GMSCs isolation and culture and all reagents used in this study were as previously described^16^. Cells from third to sixth passages were used in experiments. GMSC and human dermal fibroblasts (a cell line from American Type Culture Collection, Manassas, VA) was stained for CD14, CD26, CD29, CD34, CD39, CD44, CD45, CD73, CD90 and CD105 for phenotypes study. For each experiment, cells from one single donor were used.

### *In vitro* suppression assay of GMSC

To validate that GMSCs but not fibroblast cells have immunomodulatory function, as a big difference between these two cell types, *in vitro* suppressive assay on mouse splenic T cells was performed. Mouse splenic CD25^−^ T cells were isolated using magnetic isolation from C57BL/6 mice and labeled with CFSE using the CellTrace™ CFSE cell proliferation kit (ThermoFisher Scientific, Waltham, MA, USA) according to the manufacturer’s instructions and then stimulated with anti-CD3 (0.025 μg/ml) and irradiated (30 cGy) syngeneic non–T cells. GMSCs or fibroblasts were plated in triplicate in 96-well plates and allowed to adhere to the plate overnight. The ratio of GMSCs or fibroblast cells to mouse CD25^−^ T cells ranged from 1:1 to 1:200. Cells were cultured for 3 days and CFSE dilution of CD8^+^ T cells was tested by Flow Cytometry.

### Induction of diabetes

T1DM was induced in C57BL/6-FoxP3^gfp^ mice (male, 6–8 week old, 20–26 g body weight) using multiple low doses of STZ (Sigma-Aldrich, St Louis, MO, USA) injection. STZ was administered *via* intraperitoneal route at a dose of 40 mg/kg, dissolved in freshly made and cold 0.01 M citrate buffer (pH 4.5) within 20 minutes for 5 consecutive days. Mice were fasted for 4–6 hours prior to STZ injection and 10% sucrose water were supplied overnight after the first injection to avoid sudden hypoglycemia. To determine intervention effects, 1.0 × 10^6^ GMSCs suspended in 200ul phosphate-buffered saline (PBS) were given through *i.p*. injection on days 0, 7, 14, 21 and 28 after STZ administration. A similar dose of human dermal fibroblasts was given as a control. Meanwhile, STZ-induced model receiving 200ul PBS was considered an additional control. Non-fasting blood glucose level was monitored twice a week over the following 30 days using an Embrace one touch blood glucometer (OMNIS Health, USA). Blood glucose concentration exceeding 300 mg/dL in two consecutive daily measurements was considered diabetes. Blood samples were collected every 3 days from tail vessels and assayed for CD4^+^ and CD8^+^ Treg. Body weight was recorded every 3 days. Cages were changed every 3–4 days since mice would develop symptom of polyuria.

### Histology and immunohistochemistry

Pancreas glands were harvested on days 10 and 30. Pancreas samples were fixed overnight in 10% formalin, and embedded in paraffin. All the embedding, slicing, and H&E staining were done by the Penn State Pathology and Laboratory Medicine facility. For assessment of insulin and glucogon production in pancreas, sections were stained with anti-insulin (1:1600, AbCam63820) and anti-glucagon (1:8000, AbCam ab92517) and were revealed using anti-rabbit DAB-HRP (OMap) as secondary antibodies for visualizing, and then dying the nucleus with hematoxylin and bluing reagent. Six sections per pancreas, evenly sectioned and separated by 200μm, were stained with H&E, anti-insulin and anti-glucogon antibodies. Images were captured by Olympus microscope (Tokyo, Japan). For morphometric analysis, images of islets were traced and analyzed with the use of Image-pro-plus 6.0 software manually. Islet sizes were examined as the total islets area divided by the total number of islets. Sections were measured from at least three individual pancreases from each group by at least two different people to avoid subject bias. All islets were evaluated and the insulitis scores were determined as follows: 0, no intraislet mononuclear cell infiltration; 1, mild peri-islet mononuclear cell infiltration (granulation of <30%); 2, intraislet moderate mononuclear cell infiltration (granulation of <50%); 3, severe to massive cell infiltration (granulation of >50%)^63^.

### Tracking intraperitoneal injected GMSCs

GMSCs were labeled with CFSE using the CellTrace™ CFSE cell proliferation kit (ThermoFisher Scientific, Waltham, MA, USA) according to the manufacturer’s instructions. In brief, cells were pelleted and resuspended in PBS containing 1 μM CFSE at a concentration of 1 × 10^7^/ml and incubated for 10 min at 37 °C in a water bath. After staining, cells were washed twice in Roswell Park Memorial Institute medium (RPMI-1640) using centrifugation at 400 g for 5 min. After the final wash, a sigle dose of 2.0 × 10^6^ cells, resuspended in 200uL PBS were then injected into C57BL/6 mice. GMSCs migration and homing to different organs was examined by flow cytometry at day 3, 7, 14, and 28.

### Murine naive CD4^+^ T cell differentiation ***in vitro***

Naive CD4^+^ CD62L^+^ T cells were purified from the spleens or lymph nodes of C57BL/6 mice *via* magnetic isolation (Miltenyi Biotec). GMSCs were cocultured with naive CD4^+^ CD62L^+^ T cells (1:10 or 1:20) during their *in vitro* differentiation into T helper cells. GMSCs were allowed to adhere to the plate overnight before coculture. Naive CD4^+^ T cells were stimulated with anti-CD3 (1ug/ml) and anti-CD28 (1ug/ml) antibodies (both from Biolegend) in the presence of irradiated (30 cGy) syngeneic non–T cells (1:1), along with cytokines for Th1 or Th17 cell polarization differentiation, as previously described^16^. After 3 days in culture, differentiated cells were restimulated with PMA and ionomycin for 5 hours and brefeldin A for 4 hours. The expression of IFN-γ and IL-17 was then measured by flow cytometry.

To determine the possible mechanisms of GMSCs regulating effector T cells, GMSCs were pre-treated with kinds of solube factors, including a CD39 inhibitor (100 uM, sodium polyoxotungstate1 [POM-1]; Tocris Bioscience), CD73 inhibitor (200 uM, α, β-methylene ADP [APCP]; Sigma-Aldrich), indoleamine 2,3-dioxygenase (IDO) inhibitor (500 uM, L-1-methyltryptophan; Sigma-Aldrich) for 24 hours in 96-well, then washed twice with PBS and cells differentiation was determined as described above.

### Flow cytometry analysis

GMSCs were digested using 0.05% Trypsin-EDTA(Gibco by Life Technologies) and suspended in PBS. GMSCs and fibroblast cells phenotype identification are as previously described^16^. Mouse blood samples were collected using 0.1%EDTA(Gibco by Life Technologies) and lysis of red blood cells was processed using Red Blood Cell Lysing Buffer (Sigma-Aldrich). If lysis is incomplete, steps are repeated. Antibodies against CD4(GK1.5, PerCP/Cy5.5), CD8(53–6.7, PerCP/Cy5.5), CD28(37.51, PE), CD122(5H4, PE), CD103(2E7, PE), CD14(63D3, PE), CD26(BA5b, FITC), CD29(TS2/16, PE), CD34(561, PE), CD44(BJ18, PE), CD45(2D1, PE), CD73(AD2, PE), CD39(A1, PE), CD90(5E10, PE) and CD105(43A3, PE) were from Biolegend. Antibodies against IFN-γ(REA638, APC) and IL-17(REA660, PE) were from Miltenyi Biotec. Results were obtained on a BD FACS Calibur flow cytometry and analyzed using FlowJo. For *in vivo* IFN-γ and IL-17 detection, lymphocytes were isolated from spleen, MLN and pLN of diabetes mice on day 10 and day 30 after STZ injection, and then stimulated *in vitro* with PMA (50 ng/ml) and ionomycin (500 ng/ml) for 5 hours, with brefeldin A (10 μg/ml) (all from Calbiochem) added in the last 4 hours, and intracellular expression of IFN-γ and IL-17 was analyzed by flow cytometry.

### Statistical analyses

All data were expressed as mean ± SEM from at least three independent experiments. For comparison of more than two groups, one-way ANOVA for normally distributed data or Kruskal-Wallis for skewed data was performed. All statistical analyses were carried out using SPSS software (version 17.0). P < 0.05 was considered statistically significant.

### Data availability statements

The datasets generated and/or analysed during the current study are available from the corresponding author on reasonable request.

## Electronic supplementary material

Supplementary File
