# Supplementary material for: Human Gingiva-Derived Mesenchymal Stem Cells Ameliorate Streptozoticin-induced T1DM in mice via Suppression of T effector cells and Up-regulating Treg Subsets
Source: Sci Rep. 2017 Nov 10;7:15249. doi: 10.1038/s41598-017-14979-5 (PMC5681565; doi:10.1038/s41598-017-14979-5)
Supplement: Supplementary file 1 — Supplementary File [file 41598_2017_14979_MOESM1_ESM.pdf]

Human Gingiva-Derived Mesenchymal Stem Cells Ameliorate Streptozoticin-induced T1DM in mice via Suppression of T effector cells and Up-regulating Treg Subsets

Wei Zhang, Li Zhou, Junlong Dang, Ximei Zhang, Julie Wang, Yanming Chen, Jichao Liang, Dongqing Li, Jilin Ma, Jia Yuan, Weiwen Chen, Homayoun H. Zadeh, Nancy Olsen and Song Guo Zheng

Supplementary Figure S1

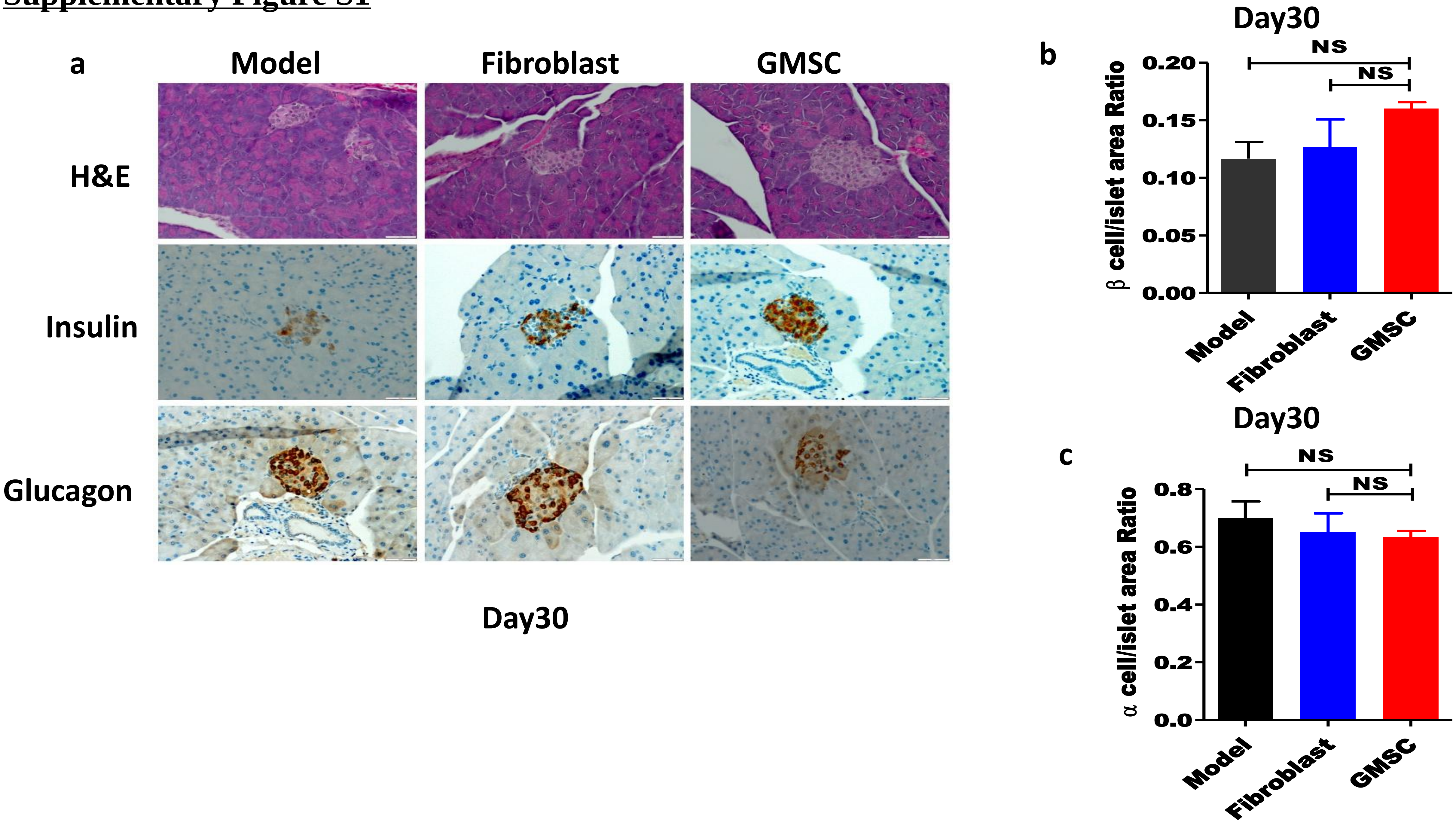

Supplementary Figure S1

GMSCs couldn’t ameliorate the severity of insulitis in mice model of T1DM on day 30.

Pancreas were harvested on day 30, fixed in 10% formalin, embedded in paraffin. Six sections per pancreas, at least three individual pancreases from each group, were evenly sectioned and separated by 200μm, stained with H&E, anti-insulin and anti-glucogon. a, Hematoxylin and eosin staining and immunohistochemistry examination of pancreas from three groups. b-c, Insulin-positive beta cells and glucogon-positive alpha cells in islets area were revealed by immunohistochemistry using the anti-insulin antibody and anti-glucogon antibody on day 30. Data are presented as the mean ± SEM from two independent experiments using at least three individual pancreases from each group. (NS: not significant)

Supplementary Figure S2

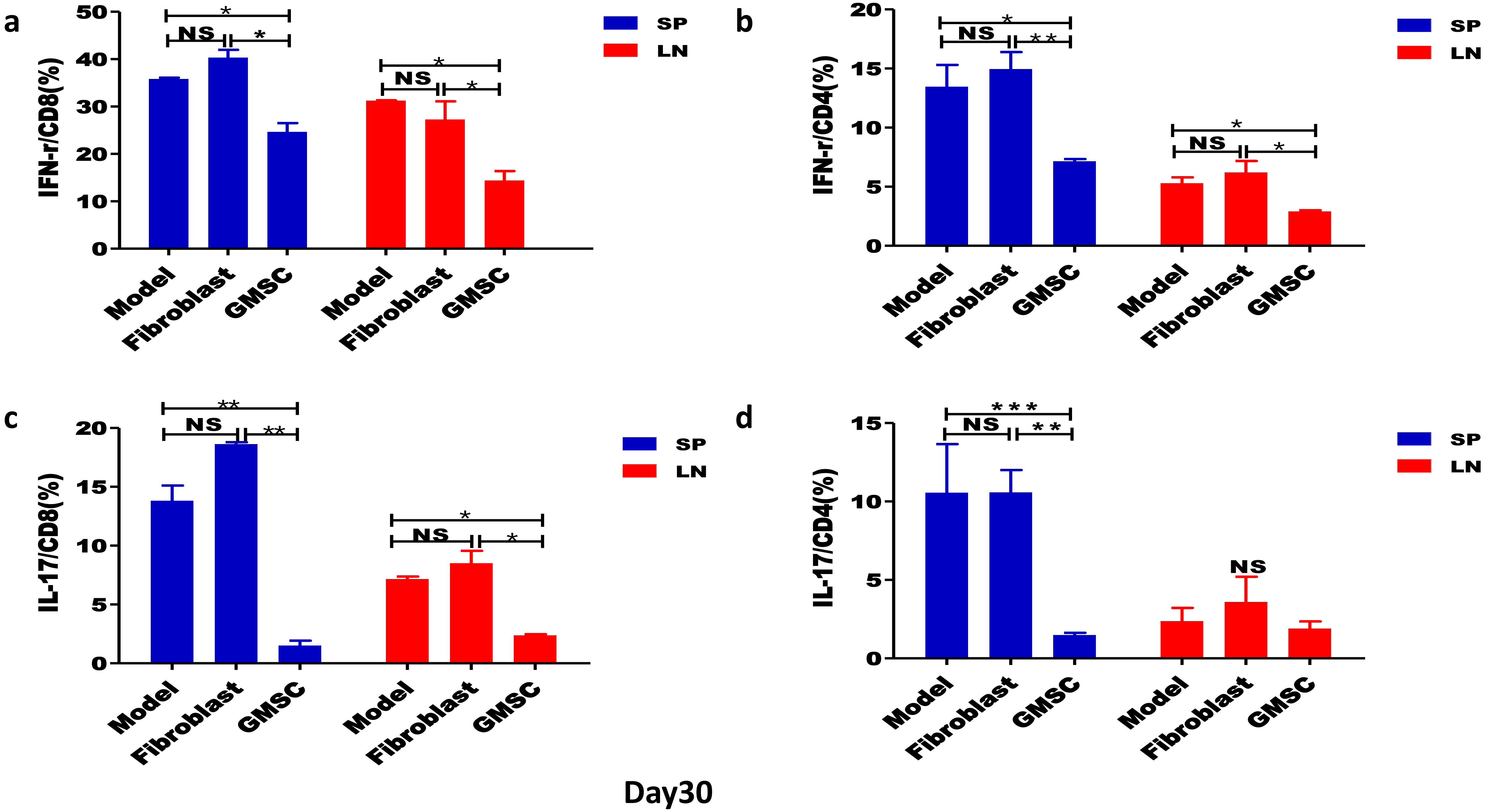

<u>Supplementary Figure S2</u>

GMSCs could also down-regulated IL-17 and IFN-γ expression in spleen and lymph nodes in STZ-induced T1DM model on day 30.

T1DM was induced using multiple low dose STZ injection and 1×10^6 GMSCs or fibroblast cells were injected into mice via *i.p.* on day 0,7,14,21,28. Spleen and other lymph nodes like MLN were harvested on day 30. Lymphocytes were isolated and then stimulated in vitro with PMA (50 ng/ml) and ionomycin (500 ng/ml) for 5 hours, with brefeldin A (10μg/ml) added in the last 4 hours, and intracellular expression of IFN-γ and IL-17 on $CD4^+$ and $CD8^+$ T cells was analyzed by flow cytometry. a-b, Expression of IFN-γ on $CD8^+$ T and $CD4^+$ T cells in spleen (blue) and LN (red). c-d, Expression of IL-17 on $CD8^+$ T and $CD4^+$ T cells in spleen (blue) and LN (red). Data are presented as the mean ± SEM from two independent experiments (n=3). *P<0.05, **P<0.01, ***P<0.01, versus the fibroblast or model group.

Supplementary Figure S3

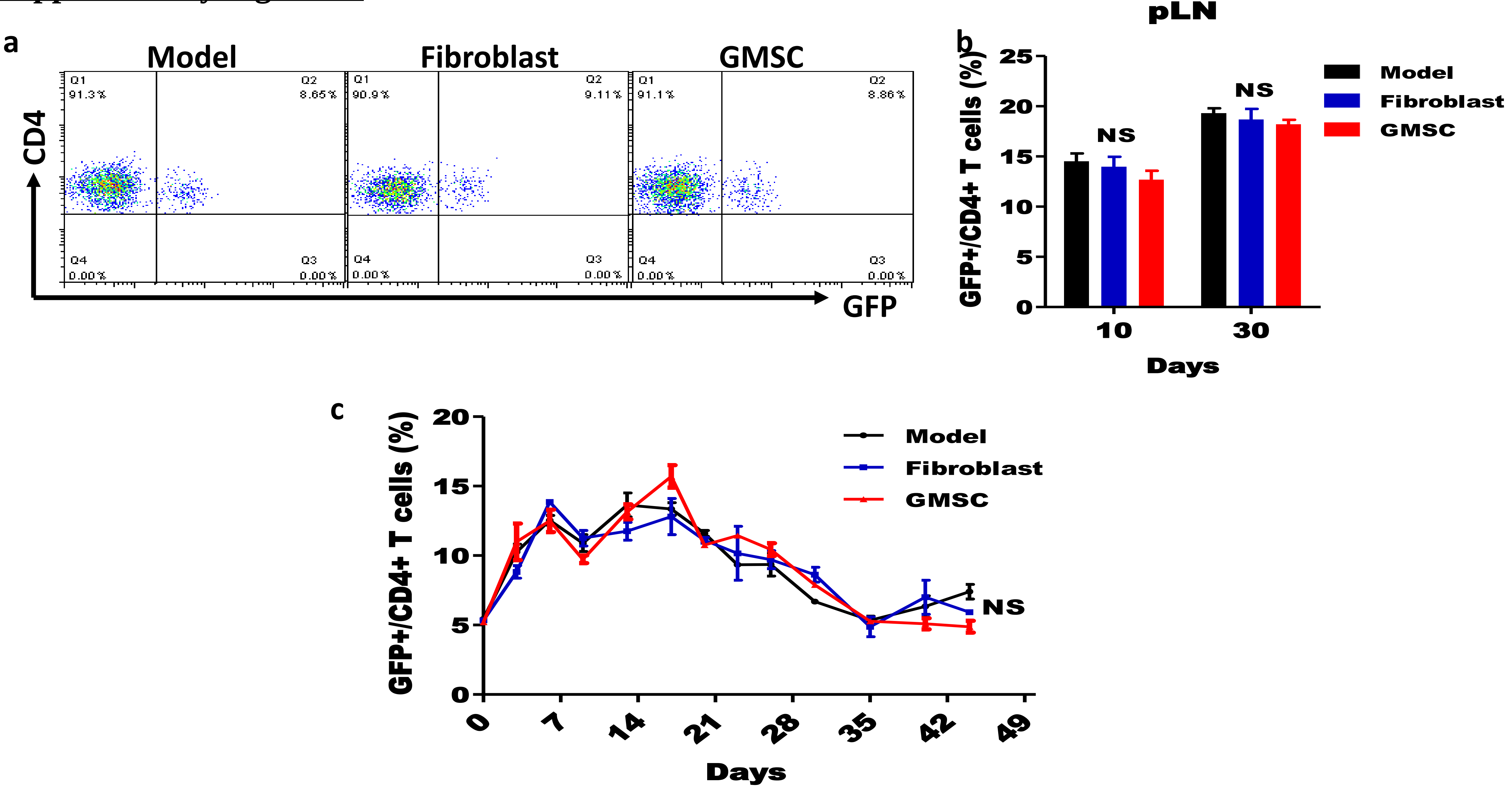

Supplementary Figure S3

GMSCs has no effect on the proportions of CD4$^+$ Tregs circulating in the peripheral blood and those residing in pLN of T1DM mice.

C57BL/6 Foxp3^{gfp} reporter mice were injected via intraperitoneal route with GMSCs (1×10^6). CD4$^+$ Foxp3$^+$ (GFP$^+$) Tregs percentage in the peripheral blood and those residing in pLN of T1DM mice were tested after GMSC injection. a, Representative flow data of CD4$^+$ Foxp3$^+$ frequency in day 30 pLN in each groups. Cells were gated on CD4$^+$ subset. b, statistics analysis of CD4$^+$ Foxp3$^+$ (GFP$^+$) Tregs percentage in pLN on day10 and day 30. Each group in each time points had three mice. Data were representative of two separate experiments and mean ± SEM of each group was shown. c, statistics analysis of CD4$^+$Foxp3$^+$(GFP$^+$) Tregs percentage in the peripheral blood dynamically after GMSCs injection. Data were representative of two separate experiments and mean ± SEM of each group (n=3) was shown.
